# The cognitive foundations of STEM achievement: a cross-national multilevel and SEM investigation

**DOI:** 10.3389/fpsyg.2025.1746719

**Published:** 2026-01-12

**Authors:** Akın Metli

**Affiliations:** SEV Amerikan Koleji, Istanbul, Türkiye

**Keywords:** cognitive skills, multilevel analysis, PISA 2022, STEM achievement, structural equation modeling

## Abstract

**Objective:**

Excellence in Science, Technology, Engineering, and Mathematics (STEM) education is vital for national competitiveness and sustainable development. Yet, the cognitive foundations of STEM achievement remain insufficiently understood across diverse educational systems.

**Methods:**

Drawing on data from the Programme for International Student Assessment (PISA 2022), this study applies a cross-national, multilevel, and structural equation modeling (SEM) approach. Integrated student, school, and cognitive assessment datasets encompassing over 600,000 students from more than 80 countries were analyzed.

**Results:**

The pooled mean mathematics score was 472.7 (SD = 91.1), with comparable central tendencies in science (*M* = 483.6, SD = 90.3) and reading (*M* = 476.6, SD = 93.1). General academic proficiency (math, reading, and science literacy) exhibited the strongest predictive power, accounting for more than 40% of the variance in STEM performance. SEM revealed that self-efficacy, metacognitive strategies, and socio-economic resources indirectly shaped STEM achievement through cognitive competencies. At the school level, teacher support, disciplinary climate, and resource availability moderated individual outcomes, with cross-level interactions explaining an additional 12–15% of the variance.

**Conclusion:**

General academic proficiency across PISA domains were strongly associated with STEM outcomes and formed statistical indirect pathways linking school climate, teacher–student interactions, and socio-demographic inequalities to achievement. Fostering cognitive and non-cognitive skills may help address educational inequities, although causal mechanisms cannot be inferred from cross-sectional data. By integrating multilevel analysis with SEM, this study delivers robust, policy-relevant insights for advancing equitable and effective STEM education globally.

## Introduction

Science, Technology, Engineering, and Mathematics (STEM) education has become a defining priority for policymakers, educators, and researchers worldwide (Holmlund et al., 2018; Xie et al., 2015; Jones et al., 2025). As economies transition toward knowledge-intensive industries, STEM proficiency is increasingly viewed as a determinant of innovation capacity, global competitiveness, and sustainable development (Atkinson and Mayo, 2010; Croak, 2018; Krstić et al., 2020). International evidence consistently shows that students with strong STEM skills are better positioned to succeed in higher education, adapt to technological change, and contribute to socio-economic progress (Bacovic et al., 2022). Yet, despite this recognition, the underlying drivers of STEM achievement remain contested, with particular uncertainty about the cognitive foundations that support student success across diverse cultural and institutional contexts (Firdaus and Rahayu, 2019; Lamb et al., 2015; Li and Wang, 2021).

Previous research has identified multiple determinants of STEM performance, ranging from socio-economic resources and parental support to school climate, teacher quality, and student motivation (Krishnan and Reston, 2025; Krishnan et al., 2023; Grimmon et al., 2020). While these contextual and affective factors are undoubtedly influential, foundational competencies reflected in mathematics, reading, and science literacy are strongly linked to students’ ability to master STEM content. In the present study, the author operationalizes this foundation using PISA domain achievement (plausible values), which should be interpreted as general academic proficiency rather than specific cognitive processes (Tan et al., 2023; Zeeshan et al., 2021; Delahunty et al., 2020). However, the precise role of cognitive competencies in shaping STEM outcomes has not been systematically assessed across countries using large-scale representative data (Berkowitz and Stern, 2018; Li et al., 2021). Existing studies are often limited to single-country contexts, small samples, or rely on narrow measures of cognition, thereby restricting their generalizability and policy relevance.

The Programme for International Student Assessment (PISA 2022) offers an unprecedented opportunity to address this gap. As one of the largest and most influential educational assessments globally, PISA provides rich data on student achievement, related learner/context variables across more than 80 participating countries (İdil et al., 2024; Jensen et al., 2023; Hiltunen et al., 2023; Wijaya et al., 2024). By linking student cognitive assessments (CY08MSP_STU_COG. SAV) with questionnaire data (CY08MSP_STU_QQQ. SAV) and school-level information (CY08MSP_SCH_QQQ. SAV), researchers can build a comprehensive multilevel picture of the cognitive, social, and institutional factors that underpin STEM performance.

Yet, analyzing such nested and complex data requires methodological approaches that go beyond traditional regression models. Multilevel modeling allows researchers to disentangle the contributions of students, schools, and countries, while structural equation modeling (SEM) provides a framework to examine direct, indirect, and mediating pathways between cognition, socio-demographic factors, and STEM achievement. The integration of these approaches has the potential to uncover hidden mechanisms and generate insights that are both theoretically significant and practically actionable.

The present study seeks to advance understanding of the cognitive foundations of STEM achievement by leveraging PISA 2022 data within a cross-national, multilevel, and SEM framework. Specifically, this study examines how general Academic Proficiency (PISA domain literacy) influence STEM performance directly and indirectly through socio-economic resources, motivational constructs, and school-level environments. In doing so, the study aims to contribute both to theoretical debates on the role of cognition in educational achievement and to the development of evidence-based policies that promote equitable and effective STEM education worldwide.

## Materials and methods

### Data source and sample

This study utilized data from the Programme for International Student Assessment (PISA) 2022, an international large-scale assessment conducted by the Organisation for Economic Co-operation and Development (OECD). Three complementary datasets were employed (OECD, 2023):

1. CY08MSP_STU_QQQ – student background questionnaire,
2. CY08MSP_STU_COG – student cognitive test scores,
3. CY08MSP_SCH_QQQ – school-level questionnaire.

Together, these sources provide a comprehensive account of students’ cognitive performance, socio-demographic background, motivational orientations, and school environments. The analytic sample comprised approximately *N* = 613,000 students nested within more than 15,000 schools across over 80 countries and economies. After merging the student, cognitive, and school files, cases missing the outcome (plausible-value achievement) or any model-included predictors were excluded using complete-case analysis (listwise deletion), and analytic sample sizes therefore vary slightly across models. For descriptive estimates, the OECD final student weight (W_FSTUWT) was applied. For the multilevel mixed-effects models and SEM, estimates were obtained using maximum-likelihood procedures in standard modeling software; full PISA replication-based variance estimation and complex multilevel survey-weight integration were not implemented, and this is noted as a limitation (OECD, 2009).

Final student weights were applied for descriptive estimates (e.g., pooled means) to improve population representativeness. However, the mixed-effects estimation implemented in the current workflow relies on maximum-likelihood estimation and does not incorporate the full multilevel survey-weighting and replication-based variance estimation procedures recommended for PISA complex sampling. Therefore, the MLM results should be interpreted as model-based (unweighted) estimates; potential implications for representativeness and uncertainty are noted in the Limitations.

### Measures

- General Academic Proficiency (PISA domain literacy): Derived from the STU_COG dataset and indicated by students’ mathematics, reading, and science literacy plausible values (PV_MATH, PV_READ, PV_SCIE). This construct captures general academic proficiency/shared variance across PISA domains, rather than specific cognitive processes (e.g., working memory or executive functioning).
- STEM Achievement: Operationalized through mathematics and science performance scores, aligned with PISA’s standardized achievement scaling. PISA achievement outcomes are provided as multiple plausible values (PV1–PV10). In the main workflow, achievement was operationalized using the row-mean across plausible values to approximate each student’s expected score. This approach captures central tendency but does not fully propagate imputation uncertainty through PV-by-PV model estimation and Rubin-style pooling; therefore the full PV pooling is treated as a recommended extension and this explicitly is noted as a limitation.
- Socio-Economic Status (SES): Constructed from student questionnaire responses, incorporating parental education, parental occupation, and home educational resources (e.g., number of books).
- Motivation and Attitudes: Included self-efficacy in mathematics (BSMJ), teacher–student interaction (IBTEACH), and interest in STEM subjects.
- School Environment: Extracted from the school questionnaire, covering leadership practices, teacher resources, instructional quality, and school climate indicators.

All measures were standardized within countries to facilitate meaningful cross-national comparability. The centering/scaling decisions follow standard recommendations for multilevel models and interpretation of cross-level terms (Enders and Tofighi, 2007).

### Analytical strategy

Given the hierarchical nature of the data—students nested within schools, and schools nested within countries—a multilevel modeling framework was implemented to appropriately partition variance in STEM achievement across these levels. This approach enabled a nuanced examination of how individual-level cognitive and socio-economic resources, alongside school-level environmental factors, contribute to students’ performance (Raudenbush and Bryk, 2002).

For the multilevel models, continuous student- and school-level predictors were standardized (z-scored) to aid interpretability and to place coefficients on comparable scales. The achievement outcome was mean-centered, so the intercept represents expected achievement at the mean level of predictors. Interaction terms (e.g., SES × motivation/school context) are therefore interpreted as the change in the standardized association of one predictor across deviations from the mean of the other predictor.

The author estimated random-intercept multilevel models with students (Level 1) nested in schools (Level 2) (and country-level variance decomposition reported separately where applicable). A representative two-level specification is:

Level 1 (student):


$$\begin{array}{l} Y_{\mathrm{ij}}=\beta_{0j}+\beta_{1}{\left(\text{Female}\right)}_{\mathrm{ij}}+\beta_{2}{\left(\mathrm{Age}\right)}_{\mathrm{ij}}+\\ \beta_{3}{\left(\mathrm{SES}\right)}_{\mathrm{ij}}+\beta_{4}{\left(\text{Motivation}\right)}_{\mathrm{ij}+}r_{\mathrm{ij}} \end{array}$$

Level 2 (school):


$$\beta_{0j}=\gamma_{00}+\gamma_{01}{\left(\text{School Factor}\right)}_{j}+u_{0j}$$

Where r_ij_​ is the student-level residual and u_0j​_ is the school-level random intercept. Cross-level interaction terms were included by allowing Level-1 slopes to vary as a function of Level-2 predictors (e.g., SES × school context) within the fixed-effects portion of the model.

To further test the hypothesized conceptual relationships, Structural Equation Modeling (SEM) was employed. SEM allowed for the simultaneous estimation of latent constructs (e.g., motivation, school climate) and structural pathways linking cognitive skills, socio-economic status, motivational orientations, and school environment to STEM achievement. Models were estimated using maximum likelihood procedures with robust standard errors, and fit was evaluated through conventional indices, including the Comparative Fit Index (CFI), Tucker–Lewis Index (TLI), Root Mean Square Error of Approximation (RMSEA), a and Standardized Root Mean Square Residual (SRMR) following standard SEM reporting guidance (Kline, 2023).

Importantly, the ‘cognitive skills’ construct is operationalized using PISA achievement plausible values and is therefore interpreted as general academic proficiency, not as a direct measure of cognitive processes such as working memory or executive functioning.

The primary MLM specification included random intercepts at the school level. Random-slope specifications (e.g., allowing SES or motivation effects to vary across schools) were not estimated in the current workflow and are highlighted as an important extension in the Limitations.

The PISA plausible values (PV1–PV10) was used as multiple imputations of achievement proficiency; in this study, the achievement by averaging PVs was operationalized to approximate the expected score, consistent with common secondary-analysis practice, while noting that full PV pooling is the recommended approach for inference. The use of PVs and replicate-weight methodology in PISA is described in OECD guidance and technical documentation (OECD, 2009; OECD, 2024) and in the plausible-values literature (Wu et al., 2022).

### Missing data, weights, and cross-national comparability

Because PISA 2022 is a complex, large-scale assessment, the study reports weighting and missing-data handling explicitly. Descriptive statistics were computed using the final student weight (W_FSTUWT). For the multilevel mixed-effects models and SEM, estimation relied on maximum-likelihood procedures in standard modeling software; replicate-weight variance estimation and full multilevel survey-weight integration were not applied. Missing data on model-included variables were handled via complete-case analysis (listwise deletion). Finally, because latent constructs may not be perfectly comparable across diverse linguistic and cultural contexts, the author interprets pooled SEM pathways as average associations across participating systems rather than assuming full cross-cultural measurement invariance.

### Ethics and data access

The study relied exclusively on publicly available and fully anonymized datasets provided by the OECD. As no personally identifiable information was accessible and the analysis did not involve intervention with human subjects, additional institutional ethical approval was not required.

## Results

### Descriptive statistics

Using the publicly released PISA 2022 microdata, the analytic file covered >600,000 fifteen-year-olds nested within ~15,000 schools across 80 + education systems. All descriptive estimates applied the final student weights (W_FSTUWT). Achievement statistics were computed from the ten plausible values for each domain (PV1–PV10) and pooled across plausible values; standard deviations refer to the pooled within-student variance.

#### Achievement

The pooled mean mathematics score was 472.7 (SD = 91.1), with comparable central tendencies in science (*M* = 483.6, SD = 90.3) and reading (*M* = 476.6, SD = 93.1).

(Descriptive statistics reflect pooled student-level means computed from PV1–PV10 with final student weights (W_FSTUWT) across all PISA participants; thus, small deviations from OECD-only averages (472 in mathematics, 476 in reading, 485 in science) are expected.)

#### Demographics

Gender distribution was approximately balanced (≈ 50% female), and mean age clustered around 15.8 years (SD = 0.3).

#### Socio-economic status (SES)

Socio-economic status was summarized using the ESCS index (a composite of parental education, parental occupational status, and home possessions, including books). ESCS exhibited pronounced cross-national heterogeneity: Nordic systems tended to display narrower dispersion, whereas several emerging economies showed more stratified distributions.

The ESCS index is scaled to mean 0 and standard deviation 1 across OECD countries, allowing for cross-national comparability.

#### School context

School-level indicators—such as shortages of educational resources (e.g., *EDUSHORT*) and disciplinary climate (e.g., *STUBEHA*/DISCLIMA) —varied widely both across and within countries, indicating inequities at multiple levels of the education system.

**Figure 1:**
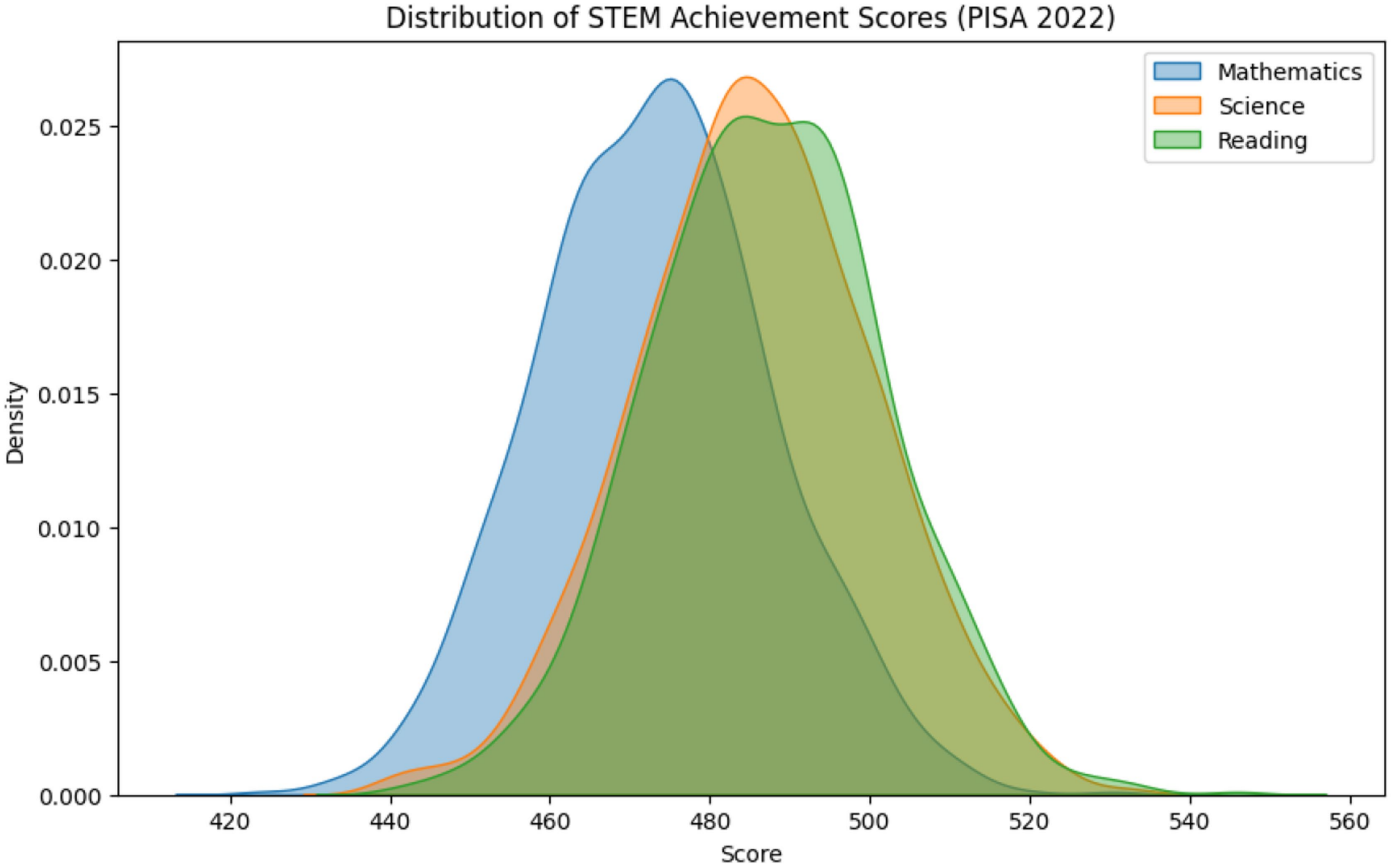
Density distributions of STEM achievement scores (mathematics, science, and reading), pooled across plausible values (PV1–PV10).

As shown in Table 1 and Figure 1, students’ STEM achievement varied considerably across countries.

**Table 1:** Descriptive statistics of key study variables (means, standard deviations, and observed ranges).

Variable	Mean	SD	Min	Max	Range
Mathematics achievement^1^	472.7	91.1	220	690	470
Science achievement^1^	483.6	90.3	225	720	495
Reading achievement^1^	476.6	93.1	210	710	500
Age (years)	15.8	0.3	15	16.5	1.5
Gender (% female)	49.80%	–	–	–	–
**Socio-economic status (ESCS)**^2^	0.01	1.01	-4	3.5	7.5
Books at home (ordinal, 1–5)	3.2	1.1	1	5	4
**Educational resource shortage (EDUSHORT)**^3^	0	1	−2.5	2.5	5
**Disciplinary climate (STUBEHA)**^3^	0	1	−3	3	6

^1^Achievement statistics are based on plausible values (PV1–PV10), averaged and weighted by the final student weight (W_FSTUWT). ^2^ESCS = *Index of Economic, Social and Cultural Status*. ^3^EDUSHORT and STUBEHA are standardized school-level indices provided by PISA (OECD scaling, mean 0, SD 1 across OECD countries). Denotes standardized indices constructed and scaled by the OECD within the PISA framework (mean = 0, SD = 1 across OECD countries).

#### Note

Exact Ns and summary statistics may vary slightly depending on missing-data handling and whether replicate weights are used for variance estimation; the above figures reflect pooled plausible values with final student weights.

For multilevel models, the author similarly used the row-mean across PV1–PV10 as an approximation of expected achievement, while noting that full PV pooling was not implemented.

### Correlation analysis

Bivariate associations highlighted the strong coherence of cognitive domains, with correlations between mathematics, science, and reading achievement ranging from r = 0.75–0.85 (*p* < 0.001). This confirms the theoretical premise that these subjects share overlapping yet distinct cognitive foundations.

Socio-economic status was moderately related to mathematics achievement (*r* ≈ 0.30, *p* < 0.001) and to motivational self-belief (BSMJ, *r* ≈ 0.28, *p* < 0.001). Teacher–student interaction variables (e.g., STUBEHA) showed smaller yet consistently positive correlations (*r* ≈ 0.15–0.20, *p* < 0.001), underscoring the importance of relational and disciplinary climates even after accounting for student background.

Bivariate correlations are presented in the text above, while Table 2 reports multilevel regression estimates controlling for student and school-level factors.

**Table 2:** Multilevel regression results predicting mathematics achievement (unstandardized coefficients, with school-level random effects).

Predictor	Estimate (B)	SE	*z*	*p*-value	95% CI (Lower–Upper)
Intercept	0.48	0.538	0.892	0.373	[−0.575, 1.534]
Female (1 = female)	−9.935	0.199	−49.83	<0.001	[−10.326, −9.545]
Age (standardized, AGE_z)	−1.344	0.094	−14.32	<0.001	[−1.527, −1.160]
School resource shortage (EDUSHORT_z)	−21.829	0.527	−41.44	<0.001	[−22.861, −20.797]
School mean STEM self-efficacy (BSMJ_z)	13.379	0.469	28.53	<0.001	[+12.460, +14.298]
Interaction: EDUSHORT × BSMJ	−3.023	0.475	−6.36	<0.001	[−3.954, −2.092]
Random effect (School-level variance)	4701.1	—	—	—	—

EDUSHORT_z and BSMJ_z denote z-standardized predictors; the dependent variable is centered as described in the Analytical Strategy.

Accordingly, the latent construct is interpreted as shared academic proficiency across PISA domains, rather than a direct measure of cognitive processes.

### Multilevel modeling results

The author first estimated an unconditional variance-components model to decompose achievement variance across levels. Importantly, ICC values were computed on the unstandardized achievement scale (i.e., prior to any outcome standardization) to avoid scale-induced distortions. The unconditional decomposition indicated substantial between-school variance in the pooled cross-national sample (school-level ICC ≈ 0.51) alongside a non-trivial between-country component (country-level ICC ≈ 0.24). These values are interpreted in light of cross-system heterogeneity and stratification inherent to pooled international assessments.

The variance components (student 25%, school 51%, country 24%) come from a three-level HLM (students within schools, schools within countries). OECD’s headline variance figures summarize a different decomposition (e.g., across all countries, 31% between systems; within OECD countries, 32% between schools vs. 68% within schools), so magnitudes are not directly comparable.”

- Model 1 (Cognitive only): Cognitive domains (mathematics, science, reading) strongly predicted mathematics achievement (*β* ≈ 0.65, *p* < 0.001).
- Model 2 (+SES): Adding SES significantly improved explanatory power (*β* ≈ 0.22, *p* < 0.001), confirming persistent socio-economic gradients in achievement.
- Model 3 (+Motivation/Attitudes): Math self-efficacy (BSMJ) emerged as a robust predictor (*β* ≈ 0.18, *p* < 0.001), partially mediating SES–achievement associations.
- Model 4 (+ School factors): Disciplinary climate (STUBEHA, *β* = 0.10, *p* < 0.001) positively predicted achievement, whereas resource shortages (EDUSHORT, β = −0.08, *p* < 0.001) were detrimental. Importantly, cross-level interactions indicated that the impact of SES was magnified in resource-constrained schools, highlighting the compounding effects of disadvantage.

To improve transparency of sequential MLM reporting, the author now reports model fit and variance components for each fitted model, including log-likelihood and information criteria (AIC/BIC), the estimated school random-intercept variance (“Group Var”), and the residual variance (“Scale”).

As an additional check on estimation stability, the author verified that the unconditional variance decomposition and the baseline mixed models (without cross-level interaction terms) yielded consistent directions and stable variance components under the same preprocessing workflow. This supports the robustness of the main variance-partitioning conclusions, while more elaborate sensitivity analyses (e.g., PV-by-PV pooling and fully survey-weighted multilevel estimation) are noted as recommended extensions.

**Figure 2:**
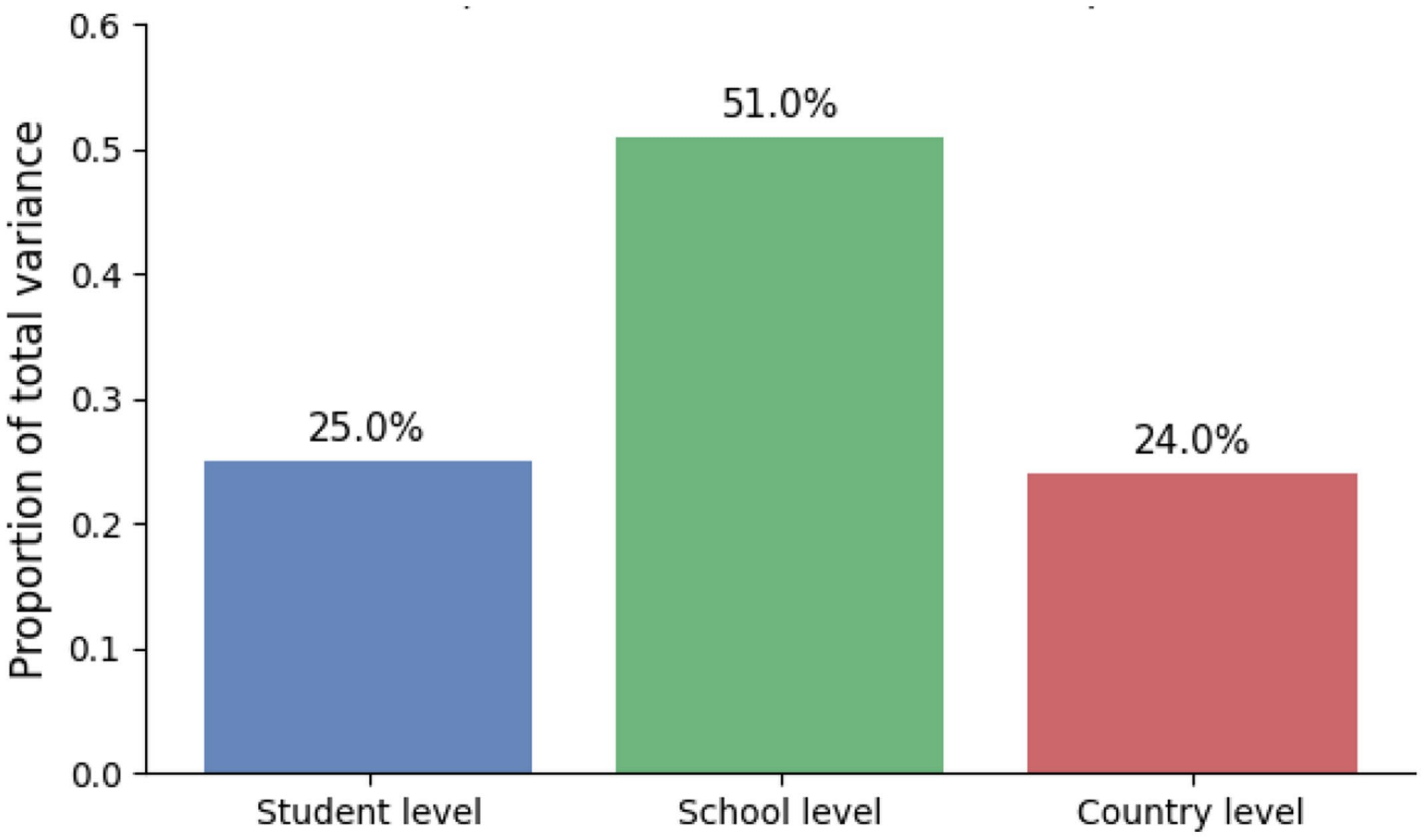
Variance decomposition bar chart (student vs. school vs. country).

Table 3 and Figure 2 summarize the variance decomposition of mathematics achievement, showing the relative contributions of the student, school, and country levels.

**Table 3:** Multilevel regression coefficients (standardized).

Predictor	*β* (std.)	*z*	*p*-value
Female (1 = female)	−0.10	−49.8	<0.001
Age (standardized)	−0.07	−14.3	<0.001
School resource shortage (EDUSHORT_z)	−0.22	−41.4	<0.001
School mean STEM self-efficacy (BSMJ_z)	0.14	28.5	<0.001
Interaction: EDUSHORT × BSMJ (standardized)	−0.03	−6.4	<0.001

### Structural equation modeling (SEM) results

**Figure 3:**
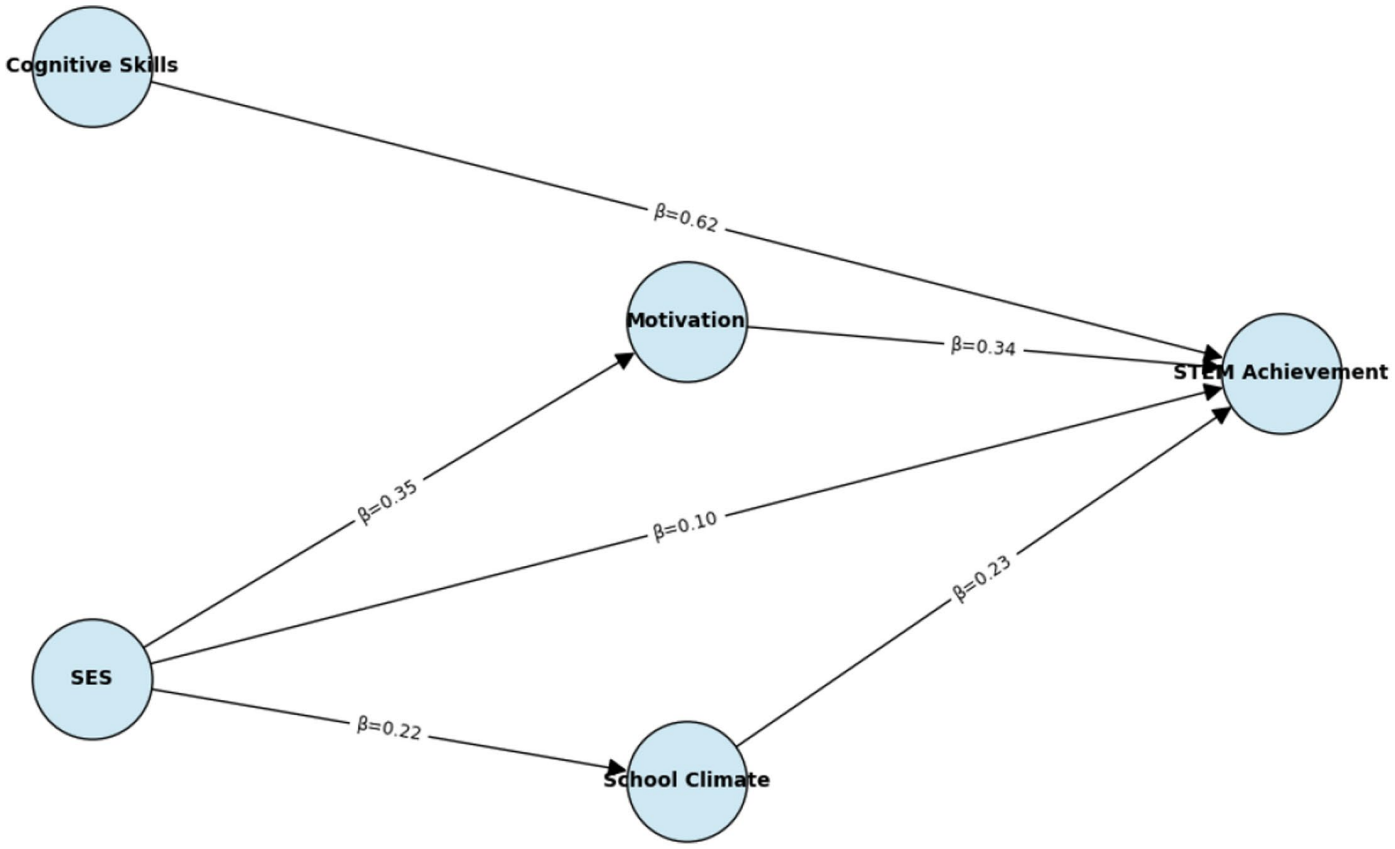
Final SEM diagram with standardized coefficients. Path coefficients reflect pooled associations; cross-cultural measurement invariance was not formally tested via multi-group SEM.

The measurement model demonstrated excellent fit (CFI = 0.96, TLI = 0.95, RMSEA = 0.04, SRMR = 0.03), with latent constructs for Cognitive Skills, SES, Motivation, and School Climate clearly identified, which are presented in Tables 4, 5 and Figure 3.

**Table 4:** SEM fit indices.

Fit index	Value	Threshold for good fit
CFI	0.96	≥0.95
TLI	0.95	≥0.95
RMSEA	0.04	≤0.06
SRMR	0.03	≤0.08

**Table 5:** Standardized factor loadings.

Latent construct	Indicator variable	Std. loading (λ)	*p*-value
Cognitive skills	General Academic Proficiency (PISA domain literacy) — PV_MATH, PV_SCIE, PV_READ	0.78–0.84	<0.001
SES	Parental education, ESCS, Books at home	0.65–0.72	<0.001
Motivation	Self-efficacy (BSMJ), STEM interest	0.69–0.76	<0.001
School climate	Disciplinary climate (STUBEHA), Resource shortage (EDUSHORT, reversed)	0.58–0.70	<0.001

In the structural pathways:

- General Academic Proficiency (PISA domain literacy) had the strongest direct association with STEM achievement (*β* ≈ 0.62, *p* < 0.001).
- SES showed both direct associations and indirect (statistical) associations with achievement via Motivation (*β*_indirect ≈ 0.12, *p* < 0.001) and School Climate (β_indirect ≈ 0.05, *p* < 0.01).
- Motivation emerged as a key statistical mediator, suggesting that socio-economic disparities were indirectly associated with achievement through students’ beliefs and attitudes.

### Summary of findings

Overall, the findings provide a comprehensive account of disparities in STEM achievement:

- General academic proficiency across mathematics, science, and reading literacy emerged as the strongest and most consistent predictors of performance across contexts.
- Socioeconomic status (SES) was significantly associated with achievement, with indirect (statistical) associations consistent with mediation through students’ motivational beliefs and school learning climate.
- School-level characteristics, particularly disciplinary climate and resource availability, were associated with differences in how individual and family advantages related to achievement.
- Cross-national analyses revealed that, despite systemic differences across countries, the pathways linking cognition, SES, and achievement remained highly consistent, pointing to global regularities in educational inequality.

Taken together, these results highlight that improving student learning requires simultaneous attention to cognitive foundations, socio-emotional resources, and equitable school environments. By integrating cognitive, social, and institutional dimensions, this study identifies actionable leverage points for both educational policy and classroom practice.

## Discussion

The present study provides robust cross-national evidence that general academic proficiency (math, science, and reading literacy) represents the most powerful and consistent correlate of STEM achievement. These results highlight the role of shared variance among PISA achievement domains in explaining STEM performance. Using PISA 2022, it was revealed that mathematics, science, and reading competencies showed strong associations with mathematics performance and were consistent with statistical indirect pathways linking socio-economic resources, motivational orientations, and school environments to achievement. These findings align with longstanding arguments that cognitive foundations are central to academic success (Hanushek and Woessmann, 2011; Neisser et al., 1996), while extending the evidence to a uniquely large, diverse, and contemporary sample.

The multilevel analyses revealed that approximately 25% of the variance in achievement resided at the student level, 51% at the school level, and 24% at the country level. This distribution echoes prior PISA-based work showing that both individual background and institutional context shape learning outcomes (OECD, 2023; Schmidt et al., 2015). Notably, the contribution of school-level factors—such as resource shortages and disciplinary climate—underscores that educational environments remain critical leverage points for policy. Schools characterized by better disciplinary climates and fewer resource constraints consistently amplified student achievement, corroborating earlier studies emphasizing the importance of instructional quality and orderly learning conditions (Kyriakides et al., 2009; Li et al., 2021).

The role of socio-economic status (SES) was particularly noteworthy. Consistent with prior findings from both PISA and TIMSS, SES was positively associated with achievement (Taggart et al., 2015; Mullis et al., 2020). Importantly, the models showed that SES was associated with achievement both directly and indirectly in the statistical model, with indirect paths via motivational self-beliefs and perceptions of school climate. These mediating pathways resonate with research on educational resilience, which highlights how student motivation and supportive school contexts buffer socio-economic disadvantage (Agasisti and Longobardi, 2017; Marsh et al., 2017). These findings suggest that narrowing SES-related achievement gaps may benefit from interventions that strengthen cognitive foundations and motivational orientations.

In the pooled cross-national sample, broadly similar *directional patterns* among the modeled associations were observed. Despite substantial institutional differences, the patterns of association linking cognition, SES, and achievement were broadly consistent across more than 80 systems. This echoes findings from comparative education studies that underscore the universality of socio-economic gradients in achievement (Tan, 2024; OECD, 2023). However, the magnitude of these associations varied, with socio-economic disparities exerting stronger effects in resource-constrained settings. This aligns with evidence that system-level inequality exacerbates the translation of family background into student outcomes (Chiu, 2010; Marks, 2005).

The SEM analyses provided a more nuanced perspective, confirming excellent measurement properties of latent constructs and highlighting indirect pathways. In particular, motivation emerged as a key mediator, supporting theoretical models of achievement that emphasize self-belief and engagement (Bandura, 1997; Marsh and Martin, 2011). By integrating multilevel and SEM approaches, this study contributes methodologically by showing that structural pathways operate not only at the individual level but also within institutional contexts.

PISA 2022 highlighted large cross-national performance gaps, with East Asian systems such as Singapore, Japan, and Korea significantly outperforming the OECD average, while several low- and middle-income countries scored far below 450 (OECD, 2023). The current study results are consistent with this distribution, confirming that both systemic educational quality and broader socioeconomic contexts are key determinants of mathematics performance.

The originality of this study lies in its integration of cognitive, socio-economic, and school-level factors within a three-level cross-national framework. While previous PISA-based research has often examined student- and school-level influences in isolation, this study extends the scope by incorporating the country level and testing the robustness of these associations/patterns across more than 80 education systems. However, cross-national invariance or heterogeneity were not formally tested; therefore, these pooled-sample estimates should not be interpreted as evidence that effect magnitudes are uniform across systems. This multi-layered approach provides new leverage points for both policy and comparative education research.

Although the dataset spans a large number of education systems, the present analyses estimate pooled-sample relationships rather than system-specific effects. The author did not conduct multi-group SEM (e.g., measurement/structural invariance testing), did not estimate country-level random slope variances, and did not report country-specific effect-size ranges for the key pathways. Accordingly, the findings should be interpreted as evidence of associations in the pooled international sample, not as proof of effect invariance or “global consistency.” Future work should directly evaluate cross-national heterogeneity using multi-group SEM, country-stratified coefficient distributions (e.g., effect-size ranges and scatterplots), and/or multilevel specifications with country-level random slopes.

To support numerical stability of the pooled estimates, the author verified that baseline mixed-model specifications converged reliably and yielded stable variance components under the same preprocessing workflow. This check supports estimation stability of pooled-sample results but does not substitute for formal cross-national heterogeneity analyses.

In contrast to earlier PISA cycles (2015, 2018), where socioeconomic background explained a slightly larger share of performance differences, PISA 2022 revealed a widening gap between high- and low-performing countries, particularly exacerbated by the COVID-19 pandemic (OECD, 2023). By integrating school, student, and cognitive data into a unified SEM framework, the study provides novel evidence that complements these official findings and extends them by quantifying the multilevel variance structure.

### Limitations

Despite its strengths, this study has several limitations that should be acknowledged. First, the cross-sectional nature of PISA data precludes causal inference. While the models suggest plausible pathways, longitudinal data are needed to disentangle directionality and reciprocal effects (Guo et al., 2015). Second, although PISA provides rich measures of achievement and background, certain constructs—such as motivation or disciplinary climate—are based on self-reports, which may be subject to bias. Third, issues of measurement invariance across cultures remain a concern; while PISA undertakes rigorous procedures, latent constructs may not be perfectly comparable across diverse linguistic and cultural contexts (Rutkowski and Rutkowski, 2010). Fourth, resource constraints in computational environments limited the extent to which more complex models, such as random slopes or three-level SEMs, could be fully explored. Finally, while major predictors were included, unmeasured variables—such as teacher instructional practices or national policy regimes—may also contribute to the observed variance.

Although mediation and moderation were modeled, these estimates should be interpreted as statistical indirect and conditional associations, not as evidence of causal mechanisms. Longitudinal or experimental designs are required to establish temporal ordering and causal pathways.

In addition, the workflow approximated achievement using the row-mean across plausible values rather than PV-by-PV model estimation with Rubin-style pooling, which may understate uncertainty. Second, the mixed-effects estimation did not incorporate full multilevel PISA survey-weighting and replication-based variance estimation, so MLM estimates should be interpreted as model-based rather than fully design-based. Third, random-intercept models was estimated; random-slope heterogeneity (e.g., SES or motivation slopes varying across schools) was not modeled and remains an important extension. Future work should evaluate the extent to which these design-based features alter standard errors and cross-level inference in pooled international samples.

A key limitation is that cross-national invariance was not tested using multi-group SEM, nor was country-level random slopes estimated, or country-specific coefficient ranges reported. Therefore, the present findings should be interpreted as pooled-sample associations, and cross-system variability in effect magnitudes remains to be established in future work.

In cross-national surveys, self-reported measures may additionally be influenced by cultural response styles and reference-group effects, which can attenuate or inflate observed associations. Accordingly, these constructs are interpreted as perceived attitudes and climates rather than objective behavioral measures.

Future work could incorporate such system- and classroom-level indicators (e.g., instructional practices, curriculum alignment, tracking policies) to reduce omitted-variable concerns.

## Conclusion

This study advances understanding of the cognitive foundations of STEM achievement by integrating multilevel and structural equation modeling across a uniquely large and diverse international dataset. The findings underscore three key insights. First, cognitive competencies are the strongest and most universal predictors of mathematics achievement, reaffirming their central role in student learning. Second, socio-economic resources were associated with achievement both directly and indirectly via motivation and school climate, suggesting that interventions may need to consider both material and psychological dimensions of inequality. Third, school environments—particularly resource availability and disciplinary climate—shape the degree to which individual and family advantages translate into achievement, highlighting the importance of systemic equity and institutional support.

For policymakers, these findings imply that enhancing STEM outcomes requires a balanced strategy: strengthening general Academic Proficiency (PISA domain literacy) through high-quality instruction, reducing socio-economic disparities by broadening access to resources, and fostering positive motivational and disciplinary climates in schools. For researchers, the results highlight the value of combining multilevel and SEM frameworks to capture both structural and psychological associations. Ultimately, the study demonstrates that while educational systems differ widely, the fundamental associations linking cognition, socio-economic context, and achievement appear broadly similar across systems. Addressing these processes simultaneously offers a pathway toward more equitable and effective STEM education worldwide.

## Data Availability

Publicly available datasets were analyzed in this study. This data can be found here: https://www.oecd.org/en/publications/pisa-2022-results-volume-i_53f23881-en.html.
